# Atrial Repolarization Waves (T_a_) Mimicking Inferior Wall ST Segment Elevation Myocardial Infarction in a Patient with Ectopic Atrial Rhythm

**DOI:** 10.1155/2018/1015730

**Published:** 2018-01-18

**Authors:** Janaki Rami Reddy Manne

**Affiliations:** Department of Hospital Medicine, Marshfield Clinic, Marshfield, WI, USA

## Abstract

We present a case of atrial repolarization waves from an ectopic atrial rhythm mimicking inferior ST segment elevation myocardial infarction in a 78-year-old male patient who presented with left sided chest wall and shoulder pain. His ischemic workup was negative, and the ST elevations completely resolved upon the resumption of sinus rhythm before discharge.

## 1. Introduction

Clinical conditions other than myocardial ischemia can affect the ST segment resulting in either ST segment elevation or depression. Pseudoinfarct patterns on electrocardiogram mimicking myocardial infarction are seen in clinical conditions like pericarditis, myocarditis, ventricular aneurysm, takotsubo cardiomyopathy, early repolarization, and cardiac memory. Atrial repolarization waves can simulate myocardial ischemia by causing ST segment elevation or depression depending upon the site of origin of the atrial impulse. Awareness and identification of pseudoinfarct patterns on ECGs is important to avoid unnecessary diagnostic interventions and treatment.

## 2. Case Presentation

**Figure 1:**
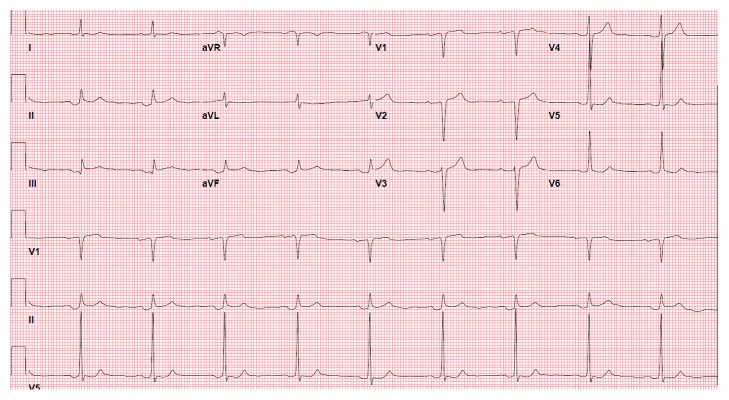
Admission electrocardiogram showing negative P waves and ST segment elevation in leads II, III, and aVF.

A man in his late 70s presented with a two-week history of constant nonexertional left sided chest pain and neck pain. His history included dyslipidemia, type 2 diabetes mellitus, and coronary artery disease that had been treated with right coronary artery stenting 10 years ago. On arrival, he had a blood pressure of 132/58 mm Hg, heart rate of 50 bpm, and oxygen saturation of 98% on room air. He received aspirin, sublingual nitroglycerin, and intravenous morphine in the emergency room which improved his chest pain. Physical examination was unremarkable except for reproducible left sided chest wall and neck pain. A 12-lead ECG obtained on admission (Figure 1) shows bradycardia with a slow ventricular rate of 50 beats/min. The P waves are inverted in leads II, III, aVF, and V4–V6 and upright in lead aVR, suggesting an ectopic atrial rhythm. The QRS duration is 90 ms, and the QT and QT_c_ intervals are 402 and 391 ms, respectively. Prominent positive atrial repolarization waves (T_a_) are seen after the QRS complexes in leads II, III, and aVF giving rise to ST segment elevation, mimicking ST elevation myocardial infarction.

**Figure 2:**
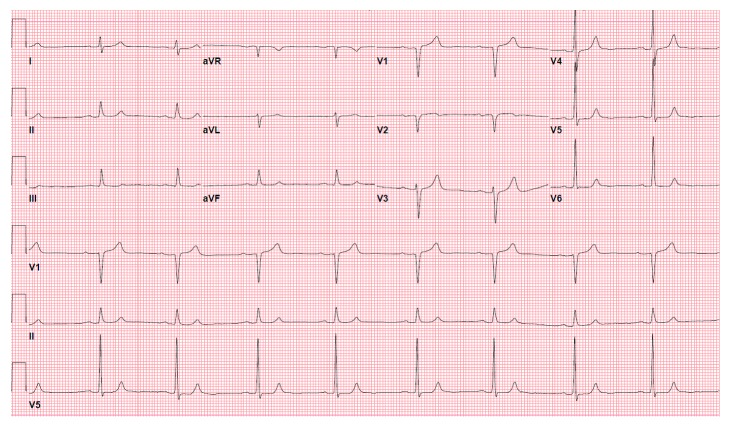
A repeat ECG showing resumption of sinus P waves and resolution ST segment elevations in the inferior leads.

Serial highly sensitive cardiac troponin I levels were less than 16 ng/L (reference range 0–45 ng/L). All the laboratory data including the inflammatory markers and electrolytes were within normal limits. A repeat ECG (Figure 2) obtained 10 minutes after his initial presentation showed resumption of sinus rhythm and complete resolution of ST segment elevation in the inferior leads.

The transthoracic echocardiogram showed normal biventricular function without any regional wall motion abnormalities. A regadenoson myocardial perfusion imaging study was negative for any reversible ischemia. The patient's chest wall and neck pain significantly improved with tramadol, and he was discharged home with a diagnosis of musculoskeletal chest wall pain.

## 3. Discussion

Atrial repolarization wave (T_a_ wave) is usually not perceptible on the ECG as it has low magnitude of 100–200 microvolts and is usually concealed by the ensuing QRS complex [1]. Occasionally, they are seen as shallow negative deflections right after the P wave in conditions with prolonged PR interval, but they are best seen in patients with complete heart block, when the T_a_ waves and QRS complexes are uncoupled [2]. In contrast to the QRS complex and T wave which under normal conditions have the same polarity, the polarity of the P wave is always opposite to that of the T_a_ wave in all leads [2]. The duration of T_a_ wave (average duration of 323 ± 56 ms) is generally 2-3 times longer than the P wave (average duration of 124 ± 16 ms) [2].

Identifying the discernible T_a_ wave and its location is relevant as it has some important clinical and diagnostic implications. In conditions with short PR interval like sinus tachycardia, the T_a_ wave can blend into the ST segment and cause ST segment depression mimicking myocardial ischemia. The T_a_ wave voltage of an inverted or retrograde P wave is always larger than that of a sinus P wave [3]. In low atrial rhythm, the atrial activation initiates from an ectopic focus rather than the sinoatrial node, and it spreads from below to upwards in the atria. The retrograde conduction to the sinoatrial node causes the inverted P waves in inferior leads while the anterograde conduction through the atrioventricular node results in normal QRS complexes. Hence, the retrograde activation of the atrium or ectopic rhythm originating in the low atrium results in a negative P wave in the inferior leads and consequently induces a positive T_a_ wave. This combination of negative P waves and exaggerated positive T_a_ waves extending into the ST segment in inferior leads can simulate an acute ST elevation myocardial infarction as seen in our patient [4]. The early repolarization pattern and atrial escape rhythm that are seen in association with increased vagal tone [5] can give rise to similar pseudo-ST elevation changes [6]; however, in our case no early repolarization changes (ST elevation at J point and terminal QRS slurring/notching) were seen on the admission or baseline electrocardiogram recordings. Exaggerated T_a_ waves can also be a cause a false positive response during treadmill stress test. The exercise-induced tachycardia increases the magnitude of the P and Ta waves and shortens the PR segment, thus shifting the atrial repolarization wave into the ST segment. In leads with prominent and upright P waves, this results in marked depression of the PR segment and the ST segment during exercise resembling myocardial ischemia [7].

As noted here, pseudoinfarct pattern with ST segment elevation or depression mimicking myocardial infarction are seen in other clinical conditions [8, 9]. Many times careful attention to the ST-T and QRS morphology, the leads involved and clinical setting in which the ST segment changes occurring are helpful in differentiating these pseudoinfarct patterns from myocardial ischemia. For example in the presence of P-R depression or prominent atrial repolarization wave, measuring the ST segment deviation relative to the TP segment results in an inaccurate measurement. To avoid errors, it should be measured in relation to the end of the PR segment, not the TP segment [10]. However, all these conditions including atrial repolarization are diagnoses of exclusion, and in some cases appropriate diagnostic testing may be necessary to exclude myocardial ischemia before establishing a definitive diagnosis.

Our case illustrates a key concept that conditions other than myocardial infarction can cause ST segment elevation. In this patient, positive T_a_ waves generated by the ectopic atrial rhythm resulted in erroneous ST elevation in inferior leads mimicking acute myocardial infarction. Misinterpretation of this ECG finding could have resulted in unnecessary treatment and cardiac catheterization.
